# Clinical Features and Outcome of Guillain-Barré Syndrome in Children 

**Published:** 2018

**Authors:** Jafar NASIRI, Mohamadreza GHAZAVI, Omid YAGHINI, Mohamad CHALDAVI

**Affiliations:** 1 Department of Pediatric Neurology, Faculty of Medicine, Child Growth and Development Research Center, Isfahan University of Medical Sciences, Isfahan, Iran

**Keywords:** Guillain-Barré syndrome, Children, Outcome, Clinical presentation, Iran

## Abstract

**Objective:**

There are no reports about the clinical presentations and outcome of Guillain-Barré syndrome (GBS) in our region, therefore, we aimed to report some mentioned findings in children diagnosed with GBS in Isfahan, central Iran.

**Materials & Methods:**

In this retrospective study, pediatric diagnosed with GBS referred to Imam Hossein Hospital, the Pediatric Referral Center of Isfahan Province, central Iran were enrolled from 2011-2014. The following data were extracted from the medical files of patients; age, gender, early signs and symptoms of GBS, neurological features, sensory and motor and autonomic involvements, sphincter dysfunction, bulbar muscle involvement, respiratory failure, cranial nerve paralysis, delay time from onset to definite diagnosis and management of GBS and the outcome.

**Results:**

Overall, 57 children with GBS aged 1-13 yr were evaluated. Frequency of GBS was significantly higher in boys than in girls (38.6% vs. 61.4%, *P*=0.01, OR=0.39). The most common clinical presentations were distal lower limb weakness (92.11%), reduced deep tendon reflex (DTR) (82.46%) and neuropathic pain (75.44%). 92.9% of patients had complete recovery.

**Conclusion:**

Distal lower limb weakness, reduced deep tendon reflex, and neuropathic pain are the main clinical presentation in children with GBS but in some patients, DTR may be normal or even exaggerated in early stage of disease. Revising the diagnostic criteria for GBS may be necessary. Most of our patients had complete recovery. The only death was due to autonomic involvement. Autonomic dysfunction could be associated with catastrophic outcome and patients with these clinical presentations need critical care.

## Introduction

The Guillain-Barré syndrome (GBS) is an acute immune-mediated polyneuropathy considered as the most common causes of acute flaccid paralysis in healthy children (1, 2). An overall worldwide incidence rate of GBS has been reported as 1 to 2 per 100000 per year (3). The rate ranges from 1.5 to 3.4 per 100000 among Iranian population (4). Although the syndrome could occur in all age groups, it predominantly affects adult population. GBS is more frequent in children aged 1-5 yr. The syndrome is more prevalent among males than females (5, 6). GBS is thought to be an autoimmune disorder that results from T and B cell activated immune response to some preceding infectious agents such as Campylobacter jejuni, Cytomegalovirus, Epstein-Barr virus, Mycoplasma pneumoniae and HIV (7, 8). Due to molecular mimicry of such infections, they could have cross-reaction with peripheral nerve components including the myelin or the axon, which consequently forms different types of GBS. Some triggering factors such as immunization, surgery, trauma, and bone-marrow transplantation could have role in the pathogenesis of GBS (7-9).

Though results of different studies from various geographical regions have reported great variability regarding the epidemiology and clinical features of GBS, the most frequent clinical presentations of GBS in children are pain, progressive muscle weakness, and reduced deep tendon reflexes. In younger children aged less than 4 yr, the most common features are pain in the legs and refusal to walk (10-12).

Understanding the clinical presentation as well as epidemiology of GBS in each population could help us in better understanding of the pathogenesis of the disease, its risk factors, and prognosis (13). Moreover, evaluating the outcome of GBD could be effective in determining the disease-related morbidity and mortality and planning appropriate therapeutic plans.

There are no reports in this regard from Isfahan, central Iran, so we aimed to report clinical findings and outcome of children diagnosed with GBS from there.

## Materials & Methods

In this retrospective study, pediatric patients diagnosed with GBS in Imam Hossein Hospital, the Pediatric Referral Center of Isfahan Province, central Iran from Sep 2011 to Sep 2014 were included.

Ethics Committee and Pediatrics Review Board of Isfahan University of Medical Sciences approved the protocol of this study.

The children were selected by census sampling method from those who fulfilled the diagnostic criteria for GBS (14) and hospitalized in the hospital. A pediatric neurologist reviewed medical files of the patients and those with complete information or the possibility of contacting the patient or his family for complete information were included. Those with missing data or non-cooperative parents were excluded.

The following information were extracted from the medical files of the patients; age, gender, early signs and symptoms of GBS, neurological features, sensory and autonomic involvements, sphincter dysfunction, bulbar muscle involvement, respiratory failure, cranial nerve paralysis, treatment modality and the outcome.

**Statistical analysis**

Data were analyzed using SPSS software (ver. 22, SPSS (Chicago, IL, USA). The continuous and categorical variables were presented as mean (SD) and n (%), respectively. The continuous and categorical variables between and within studied groups were compared using Student’s *t*-test and Chi-square test, respectively. The level of statistical significance was set at *P*<0.05.

## Results

Overall, 57 children with GBS within the age of 1-13 yr were evaluated. Characteristics of all studied population, frequency of different clinical presentations of GBS and their outcome are presented in Table 1. Frequency of GBS was significantly higher in boys than in girls (38.6% vs. 61.4%, *P*=0.01, OR=0.39). Male to female rate in the occurrence of GBS was 1.59 (35/22).

GBS was more prevalent among children aged less than 10 yr old (*P*<0.001, OR=27.19). The most common clinical presentations were distal lower limb weakness (92.11%), reduced deep tendon reflex (82.46%) and neuropathic pain (75.44%).

In our study, 7(12.28%) and 3 (5.26%) of the patients had normal and increased deep tendon reflex in initial stage of disease respectively. All of them developed hyporeflexia or areflexia in later stage of their illness.

**Figure 1:**
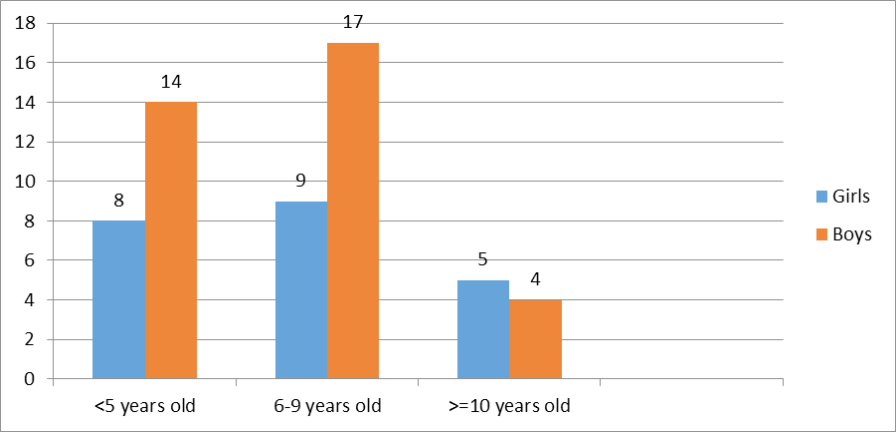
Frequency of Guillain-Barré syndrome according to gender and age groups

**Figure 2:**
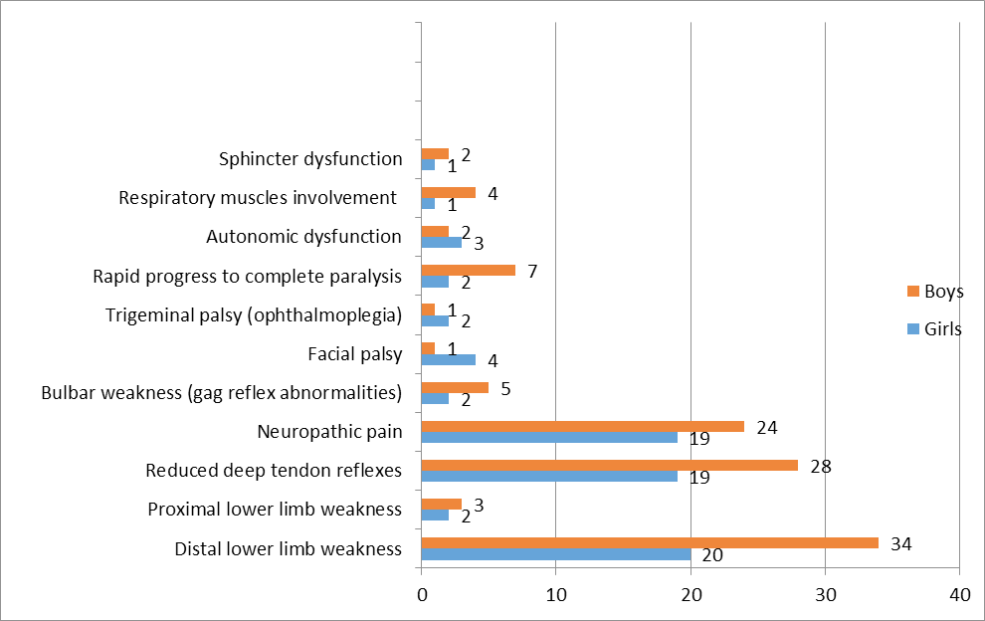
Distribution of different clinical feature of Guillain-Barré syndrome in boys and girls

**Table 1:** Characteristics and frequency of different clinical presentations of Guillain-Barré syndrome in affected children

**Variables**	
**Age(yr)**	5.78(3.19)
**Age groups [n(%)]**	
**-<5 years old** **-5-9 years old** **->=10 years old**	22(38.6%) 26(45.6%) 9(15.8%)
**Sex [n(%)]** **Girls/boys**	22(38.6)/35(61.4)
**Time between onset to definite diagnosis of GBS(days)**	2.07(1.59)
**Clinical presentations**	
***lower limb weakness***	
**-Distal** **-Proximal**	52(91.22) 5(8.77)
***Deep tendon reflexes (DTR)***	
**-Reduced** **-Normal** **-Increased**	47(82.46) 7(12.28) 3(5.26)
***Neuropathic pain***	43(75.44)
***Cranial nerve Involvement*** ***-*** **Bulbar weakness (gag reflex abnormalities)** **-** ** Facial palsy** **-ophthalmoplegia**	15(26.31) 7(12.28) 5(8.77) 3(5.26)
***Rapid(<3 days during hospitalization) progress to complete paralysis***	9(15.79)
***Autonomic dysfunction***	5(8.77)
***Respiratory muscles involvement and mechanical ventilation***	5(8.77)
***Sphincter dysfunction***	3(5.26)
**Outcome**	
**Complete recovery**	53(92.9%)
**Incomplete recovery**	3(5.3%)
**Death**	1(1.8%)

Frequency of GBS according to gender and age groups are shown in Figure 1. Though the frequency of GBS syndrome occurrence was higher in boys than girls in younger age group, the differences between girls and boys were not statistically significant (*P*=0.51).

Distribution of different clinical feature of GBS in boys and girls are shown in Figure 2. Frequencies of different clinical features of GBS were not significantly different between girls and boys. Fascial palsy was more prevalent among girls (*P*=0.07) and respiratory muscle involvement was more frequent presentation among boys (*P*=0.07).

According to our treatment protocol for patients with GBS, 53 patients with significant motor disability (nonambulant) had been treated with IVIG. Four cases with mild symptom preserved ambulation were discharged without any treatment.

Seven patients (5 of them needed prolonged mechanical ventilation) had received second course of IVIG. Two of this patient with severe disability were treated with plasmapheresis after first course of IVIG and again received IVIG after plasmapheresis. 

Electrophysiological study was performed for 49 patients. Eighteen patients had axonal (7 sensory-motor and 11 motors) and 31 demyelinating type of polyneuropathy. There was no significant correlation between initial presentation and results of electrophysiological study (*P*>0.05). For ethical considerations, ethical approval was issued by the Research the Isfahan University of Medical Sciences (93-393586) and relevant authorities for this study

## Discussion

We reviewed clinical presentations and outcome of all pediatric patients with GBS referred to our center. GBS is more common in boys and in children aged less than 10 yr old. The most common clinical presentations were distal lower limb weakness, reduced deep tendon reflex, and neuropathic pain. A high proportion of patients had complete recovery.

In our study, most of the patients were aged less than 10 yr old. Though patients in the 6-9 yr age group were higher than those aged less than 5 yr old, the difference was not significant. Some studies in this field have reported higher rate of GBS in children younger than 5 yr old (15, 16), while others reported higher rate in children aged 5-10 yr (17, 18). Almost all of the studies have reported lower occurrence of GBS in children older than 10 yr old (15-18). Comparing with reports from different region of Iran, mean age of our patients was similar to previous studies in Iran (4, 19, 20).

Young children are more prone to the infections contributing to the pathogenesis of GBS. Another explanation is possibly of higher susceptibility of myelin to demyelination in younger children (17).

According to previous studies, GBS frequently affect boys more than girls. Different rate of male to female ratio has been reported for GBS. Our reported ratio was 1.59. It was similar to that reported by other studies (4, 17-20).

The most common clinical presentations were distal lower limb weakness, reduced deep tendon reflex, and neuropathic pain, similar to another study (21), and those reported in Mashhad, Tabriz, and Tehran in Iran (4, 19, 20).

The proportion of children with atypical clinical presentation of GBS was high with a rate of 24.2% (19). 

In previous studies in Japan and Singapore, 13% and 10% of the patients with GBS had normal or increased DTR respectively (22, 23). In our study, 7(12.^29^) and 3(5.^26^) of the patients had normal and exaggerated deep tendon reflex in initial stage of disease respectively. All of these patients developed hyporeflexia or areflexia in later stage of their illness. This finding suggests revising the diagnostic criteria for GBS. 

Reported range for cranial nerve involvement is 30%-46% (4, 21, 24). In our study, 26.3% of patients had cranial nerve involvement, bulbar weakness, followed by facial nerve and trigeminal palsy.

Our reported rate of cranial nerve palsy was similar to that reported in Korea (25), India (17) and Iran (19). It was lower than that of Tabriz (4) and Mashhad (21), but was higher than that of France (18).

The feature of cranial nerve palsy with predominance of bulbar nerve followed by facial nerve was similar to that reported in Mashhad (24) and Tabriz (4). Most studies reported that the involvement of facial nerve was more common than others (26).

Autonomic dysfunction is mainly manifested as abnormal sweating, sinus tachycardia, blood pressure instability, and pupillary abnormality (18). The frequency of autonomic dysfunction in our study was 8% that was lower than the rates reported by previous studies in both Iran and other countries (10, 20). The outcome of one case with this presentation was death, the only death case among in our patients. He had tachycardia, abnormal sweating, and blood pressure instability. The patient died due to recurrent uncontrollable cardiac arrhythmias. 

The risk of respiratory muscles involvement requiring ventilatory support is lower in children than adults (27). In our study, 8% of patients received ventilator support because of respiratory insufficiency. The rate was reported to be 35%, 27%, 12% and 10.5% in India (17), Taiwan (28), France (18) and Tabriz (4), respectively. In a prospective multicenter study in Germany, 13% required artificial ventilation (29).

Sphincter dysfunction was reported in 5% of our studied population. The rate was 15.2% in the study in Tehran (19).

The prognosis of GBS in children is better than adults (18). In general, it has shorter clinical course with higher rate of complete recovery. Reported mortality rate for pediatrics GBS has been reported as 1%-2% (30). In this study, 92% of patients had complete recovery with a 1.8% mortality rate. Our findings were similar to most of the reported studies in France (18), Tabriz (4) and India (17). No death related to GBS was reported in Mashhad (20).

In this study, 5% of patients had incomplete recovery that was similar to that reported in India (17). The rate was lower than that reported (19) in Tehran with a rate of 27%. The main cause is higher rate of patients with a typical clinical presentation.

The limitations of this study were lower sample size and missing data regarding the antecedent illness preceding GBS.

**In conclusion, **clinical presentation of GBS in majority of the patients is similar to previous studies but despite current diagnostic criteria for GBS, in a significant portion of the patients, DTR may be normal or even increased in early course of disease. This finding suggests revising the diagnostic criteria for GBS. Most of the patients had favorable prognosis. The only death was due to uncontrollable cardiac tachyarrhythmia in a patient with autonomic nervous system involvement. Autonomic dysfunction could be associated with catastrophic outcome and patients with these clinical presentations require critical care.

Results could be utilized as baseline data for better understanding of the characteristics of GBS in children and consequently better management of the disease as well as development of prognostic modeling score for our patients. It is also recommended to design prospective study in this field to achieve more results that are accurate.
